# Noninvasive Assessment Methods for the Therapeutic Effect Following Facial Rejuvenation

**DOI:** 10.1111/jocd.70482

**Published:** 2025-10-11

**Authors:** Shasha Zhao, Meng Wang, Xiaodong Lai, Yan Yan

**Affiliations:** ^1^ Department of Dermatology Plastic Surgery Hospital, Chinese Academy of Medical Sciences and Peking Union Medical College Beijing China

**Keywords:** biophysical parameters, efficacy evaluation, facial rejuvenation, imaging techniques, subjective assessment tools

## Abstract

**Background:**

As the demand for facial rejuvenation continues to rise, monitoring the response to therapy is worthy of attention. However, a standardized system for assessing therapeutic effects is currently lacking. This study aims to review the existing noninvasive efficacy evaluation tools for facial rejuvenation and analyze their advantages and disadvantages.

**Methods:**

An extensive literature search was conducted in PubMed to identify articles on efficacy evaluation tools for facial rejuvenation published between 1995 and 2024.

**Results:**

We classify the available measurement tools as subjective assessment methods, imaging techniques, and biophysical methods. Most subjective scales are based on clear definitions and standardized photographs, which are evaluated by dermatologists. Various scales focusing on different clinical features are continuously being developed, making it challenging to compare study outcomes. Imaging techniques are generally more precise and accurate. The information collected by these techniques is more convenient to store, search, and analyze, indicating their significant potential for monitoring efficacy. However, the high cost of equipment and complexity of operation may limit the application. Biophysical parameters can indirectly measure the severity of skin aging, but their repeatability and accuracy require further validation.

**Conclusion:**

This review offers a comprehensive overview of the existing efficacy evaluation methods for facial rejuvenation and discusses pertinent details. There is no single best assessment method. When selecting outcome measures in clinical practice or scientific research, several factors must be considered, including therapeutic objectives, convenience, and accuracy.

## 1 Introduction

Skin aging is a highly complex process that results in alterations to both the structure and function of the skin. Intrinsic skin aging is closely associated with cellular aging, telomere shortening, mutations in mitochondrial DNA, oxidative stress, and hormonal changes. Chronic ultraviolet‐induced deterioration mainly contributes to extrinsic skin aging [1]. Aging skin is characterized by reduced elasticity, decreased collagen content, deep wrinkles, increased pigmentation, and a compromised skin barrier [2]. These changes can significantly impact an individual's appearance and negatively affect perceived attractiveness, self‐esteem, and overall quality of life.

Thus, the demand for facial rejuvenation is increasing rapidly. Topical agents, laser therapy, energy‐based treatments such as radiofrequency, and surgical interventions are widely used in clinical practice [3]. However, there are no standardized clinical evaluation systems in place to monitor their efficacy, making it difficult to compare the improvements among different treatment options.

Generally, the tools available to assess skin rejuvenation can be classified into subjective and objective methods. Subjective methods mainly rely on visual comparisons of the skin before and after treatment. Objective methods primarily encompass imaging techniques and the measurement of biophysical parameters. Imaging techniques are continuously evolving and can provide increasingly detailed information regarding treatment outcomes.

The current literature aims to provide an overview of the available noninvasive measurement techniques for assessing the efficacy of anti‐aging therapies and to compare their advantages and disadvantages.

## 2 Subjective Assessment Methods

### 2.1 Wrinkle Assessment

Visual wrinkles have the most significant impact on appearance. Most anti‐aging treatments have been developed to reduce the severity of wrinkles. Consequently, wrinkle assessment tools are commonly utilized in practice (Table 1).

**TABLE 1:** Scales widely used to assess wrinkle severity.

Scale	Details
Fitzpatrick Wrinkle Classification System (FWCS)	I = fine wrinkles; II = fine to moderate depth wrinkles, moderate number of lines; III = fine to deep wrinkles, numerous lines, with or without redundant skin folds
Modified Fitzpatrick Wrinkle Scale (MFWS)	0 = no wrinkle; 0.5 = very shallow yet visible wrinkle; 1 = fine wrinkle; 1.5 = visible wrinkle and clear indentation; 2 = moderate wrinkle; 2.5 = prominent and visible wrinkle; 3 = deep wrinkle
Wrinkle Severity Rating Scale (WSRS)	1 = no visible nasolabial fold, continuous skin line; 2 = shallow but visible nasolabial fold with a slight indentation, minor facial feature, implant is expected to produce a slight improvement in appearance; 3 = moderately deep nasolabial fold, clear facial feature visible at normal appearance but not when stretched, excellent correction is expected from injectable implant; 4 = very long and deep nasolabial fold, prominent facial feature, < 2 mm visible fold when stretched, significant improvement is expected from injectable implant; 5 = extremely deep and long nasolabial fold, detrimental to facial appearance, 2‐4 mm visible V‐shaped fold when stretched, unlikely to have satisfactory correction with injectable implant alone
Lemperle Wrinkle Assessment Scale	0 = no wrinkle; 1 = just perceptible wrinkle; 2 = shallow wrinkle; 3 = moderately deep wrinkle; 4 = deep wrinkle, well‐defined edges; 5 = very deep wrinkle, redundant fold

In 1996, Fitzpatrick and his colleagues proposed the Fitzpatrick Wrinkle Classification System (FWCS) to assess the curative effect of laser treatment [4]. Common features evaluated included wrinkling, elastosis, dyschromia, and wrinkle depth. Scores ranged from 1 to 9, and severity was defined as three classes: I = fine wrinkles, II = fine to moderate depth wrinkles, and III = fine to deep wrinkles. This system is widely utilized in clinical trials to evaluate the severity of perioral and periorbital wrinkles. The Modified Fitzpatrick Wrinkle Scale (MFWS) was developed as an adaptation of the FWCS specifically for assessing nasolabial folds [5]. Compared to reference photographs, scores range from 0 to 3 (0 = absent, 1 = fine, 2 = moderate, 3 = severe). Additionally, three intermediate scores (0.5, 1.5, 2.5) can be used without reference photographs.

Wrinkle Severity Rating Scale (WSRS) was specifically designed to measure nasolabial folds based on their length and apparent depth. A set of three reference photographs was used to define five grades [6]. Previous research has demonstrated that the WSRS is effective in assessing the outcomes of soft‐tissue augmentation treatments. Lemperle proposed a six‐grade classification system in 2000 to measure wrinkle depth [7]. The scale considered wrinkles in 12 facial regions. In 2010, Lawrence Buchner et al. refined the tool for evaluating nasolabial folds using standardized photographic techniques [8].

### 2.2 Photoaging Assessment

Glogau photoaging classification was developed to assess photoaging, particularly the severity of wrinkles. The severity was divided into four types: type I (no wrinkles), type II (wrinkles in motion), type III (wrinkles at rest), and type IV (only wrinkles) [9]. Global scores for photoaging (GSP) evaluate the overall severity of facial skin aging using a five‐point scale [10]. Factors such as skin roughness, dyspigmentation, lines, and the number of affected areas were taken into account. Garg et al. developed a classification and scoring system based on drooping and wrinkles in various areas of the face, which has been validated for Indian participants [11]. This system can be used to categorize patients and assess treatment efficacy.

### 2.3 Global Assessment

By comparing the standardized photographs before and after treatment, physicians and patients can evaluate overall improvement using the Global Aesthetic Improvement Scale (GAIS). This instrument consists of a five‐point scale: 1 = very much improved, 2 = much improved, 3 = improved, 4 = no change, 5 = worse [12].

The FACE‐Q Aesthetics module is a validated patient‐reported outcome measure designed to assess patients' satisfaction with their facial appearance, quality of life, adverse effects, and satisfaction with the process of care. This scale consists of a series of independently functioning checklists that evaluate different regions of the face [13].

Investigator Global Assessment (IGA) and Physician Global Assessment (PGA) are the most common global assessment tools. Patients can also report their skin condition using the Visual Analogue Scale (VAS) questionnaire, which considers skin elasticity, hydration, radiance, firmness, wrinkles, and overall feel. Additionally, certain scales that assess quality of life and self‐esteem can indirectly measure improvement in facial appearance [14].

### 2.4 Others

Alexiades‐Armenakas described a comprehensive grading scale for rhytides, skin laxity, and texture in 2006, primarily focusing on skin laxity [15]. The Allergan Skin Roughness Scale is a validated and reliable tool for physicians to assess midface skin roughness [16]. Regarding wrinkles in different facial regions, several scales have been adapted to evaluate wrinkles in each area, such as the Allergan Infraorbital Hollows Scale and the Allergan Forehead Lines Scale [17, 18]. Some new assessment systems have been developed to account for the varying characteristics of skin aging across different races [19].

## 3 Imaging Techniques

### 3.1 Dermoscopy

Dermoscopy is a noninvasive and reproducible tool for skin visualization, allowing for the detection of subtle and early signs of aging. This technique is commonly used for diagnosing and monitoring skin disease. Dermoscopic photoaging scale (DPAS) consists of 11 criteria, which are assessed by scoring different facial areas: left malar, right malar, forehead, and chin. Each area is evaluated with a yes or no response, resulting in a total score that ranges from 0 to 44 [20, 21].

### 3.2 Ultrasonography (US)

The visualization of US is based on the distinct reflection patterns of various tissues. Studies have shown that epidermal thickness increases following anti‐aging therapy [22]. Concurrently, the thickness of the subepidermal low echogenicity band (SLEB) increases linearly with age, serving as a valuable parameter for assessing photoaging [23]. Variations in skin echogenicity are related to structural changes in aging skin. For instance, Low Echogenic Pixels can objectively assess the levels of skin hydration, inflammatory processes, solar elastosis, and collagen degeneration, while Medium and High Echogenic Pixels can quantify the structures of collagen, elastin fibers, and microfibrils [24]. High‐frequency ultrasound skin imaging analysis (HFUS) offers greater precision [25]. However, it is essential to acknowledge that US imaging somewhat depends on the operator's skill. Additionally, using US, specific epidermal structures and the epidermis‐dermis junction can be challenging to analyze.

### 3.3 Reflectance Confocal Microscopy (RCM)

RCM is a noninvasive technique that provides real‐time images of the skin, allowing for the assessment of cellular features in the epidermis and dermis with nearly histological resolution [26]. In 2011, Longo et al. evaluated the facial skin in volunteers of various ages by RCM and delineated the skin changes of specific epidermal and dermal features over time, such as the presence of irregularly shaped keratinocytes, epidermal atrophy, and curled elastotic fibers [27]. In 2014, Haytoglu et al. supplemented other confocal parameters related to skin aging from 120 volunteers, including increased mean furrow depth [28]. However, the reticular dermal changes cannot be assessed due to the limited depth of laser penetration.

### 3.4 Optical Coherence Tomography (OCT)

OCT images the skin, especially dermal collagen, using light‐based interferometry to create digital images of photoaging skin. Key findings from OCT imaging of photoaged skin include epidermal atrophy, loss of papillary dermis, and dermal mottled texture [29]. OCT offers excellent reliability between different observers, and the data can be stored for future blinded analysis, aiding comparisons in clinical trials [30]. However, the penetration depth of OCT is a notable limitation, which may be addressed by using other acousto‐optic imaging systems concurrently.

### 3.5 Skin Imaging Analysis Instruments

The Antera 3D utilizes a technology that reconstructs the skin in 3D through shape from shading and photometric stereo measurements, using multiple images taken under various light sources across seven wavelengths in the visible spectrum [31]. Messaraa et al. have verified its reliability in monitoring the effectiveness of cosmetic products [32].

The DermaTOP system employs interference fringe projection profilometry to collect 3D information on skin topography, such as profile roughness parameters and wrinkle object parameters. Lagarde et al. have proved its accuracy and repeatability, so it is widely used in the quantitative analysis of skin improvement [33]. The AEVA‐HE system can quantify the major characteristics of wrinkles, based on similar technology [34].

The VISIA skin analysis system uses standard incandescent light, ultraviolet, and cross‐polarized light, generates a series of high‐resolution images, and analyzes the overall skin conditions rapidly, such as wrinkles, texture, pores, and visible wrinkles. This device is widely used in clinical practice and research [35, 36]. Other facial skin measurement systems, such as VisioFace and Janus‐III system, operate on similar principles and can quantify skin characteristics [37].

### 3.6 Computer‐Aided Photo Measurement

AutoCAD software is a mainstream drawing tool that can measure the length or area of a specific region. For example, a reduction in area defined by fixed points can indicate clinical results consistent with neck and submental lifting. Similarly, eyebrow height and nasolabial fold length measurements can reflect skin lifting and tightening [38].

Stephens Wrinkle Imaging using Raking Light (SWIRL) utilizes raking light optical profilometry to analyze facial wrinkles across multiple regions [39]. When combined with Image Pro Plus software, it can automatically quantify five parameters of wrinkles: number, length, width, area, and depth.

Wrinkle gray scale (WGS) focuses on the depth of wrinkles, which can numerically measure the difference in brightness between the wrinkles and the surrounding skin [40]. The more pronounced the wrinkle, the higher the gray value appears. A similar method can also be employed to assess the severity of baggy lower eyelids [41].

## 4 Biophysical Methods

The characteristics of aging skin include reduced elasticity, decreased collagen content, increased pigmentation, and dysfunctions in the epidermal barrier. Consequently, fluctuations in biophysical parameters can indirectly quantify improvements following aesthetic treatments.

The change in skin hydration is related to skin texture and roughness, such as transepidermal water loss (TEWL) and water content in the stratum corneum. TEWL is always measured by the closed‐chamber VapoMeter and the open‐chamber Tewameter. The Corneometer can gauge skin electrical capacitance to determine water levels in the stratum corneum. Skin firmness can be assessed with the Cutometer, which measures skin deformation by drawing it into a hollow aperture by applying negative pressure or suction. A higher score from the Cutometer indicates greater skin elasticity. Skin pigmentation is usually measured by reflectance spectrophotometric methods and expressed as L*a*b* values or melanin and erythema indices. The Mexameter quantifies erythema and melanin using narrow‐band reflectance measurements based on the differential absorption of light by melanin and hemoglobin at specific wavelengths. Skin pH and sebum level can be assessed by skin pH meter and Sebumeter, respectively [42, 43].

## 5 Discussion

With the rapid advancement of facial rejuvenation techniques, there is a growing need for reliable and accurate methods to detect subtle improvements following anti‐aging treatments. However, achieving a consistent level of monitoring for therapeutic responses remains challenging. Most methods can only measure one or a few features in the complex environment of aging skin. Only a suitable assessment tool can represent treatment outcomes. In this review, we summarize published assessment methods and discuss their advantages, disadvantages, and technical challenges.

Subjective assessment tools are the most commonly utilized in clinical practice. These tools evaluate improvement or severity by comparing current photographs with baseline or standardized images. A clear and straightforward definition for each grade serves as a reference point. Both physicians and patients can easily engage in the evaluation process, as it is not constrained by time or location. The results are based on a combined impression rather than specific metrics. The characteristics of aging facial skin are diverse, and different techniques prioritize various aspects. For example, the FWCS focuses on generalized wrinkling and elastosis rather than the depth of specific wrinkles, which is more critical for measuring the effectiveness of injectable fillers [6]. Therefore, it is essential to choose an appropriate scale according to specific therapeutic goals. Global assessments can measure the effectiveness of all treatments, providing intuitive and easily understandable outcomes.

High‐quality and standardized photography is also crucial for subjective assessment, ensuring reliable and reproducible comparisons before and after treatment. This documentation facilitates the longitudinal tracking of patient progress and enables clinicians to detect subtle improvements with greater accuracy. Practitioners with extensive experience play a key role in capturing and interpreting the photos. However, minor improvements may not be captured by a simple grading system due to its limited scale. Additionally, the increasing number of modified grading systems complicates the comparison of outcomes across studies.

Objective assessment methods are more beneficial for research, yielding more objective and reliable results. Portable dermoscopy can be utilized in various settings and can assess all regions of the face. High‐frequency ultrasound has a penetration depth of approximately 15 mm with a limited resolution, making it difficult to detect slight tissue variations. In contrast, RCM offers high resolution but only penetrates to a depth of 0.2 mm. OCT may provide a better balance between penetration depth and resolution [29]. Antera 3D has demonstrated superior sensitivity and repeatability in the global assessment of skin features like color, wrinkle, and pore [44]. In addition, this equipment has the advantage of being portable, user‐friendly, and capable of analyzing larger skin areas. Computer‐aided measurement is quicker and more straightforward, directly reflecting skin lifting. The SWIRL method and WGS analysis, which employ optical profilometry, can quantify the severity of facial wrinkles before and after treatment. Analysis software like Image J is free and user‐friendly [40].

The costs associated with imaging techniques, including equipment, training, operational, and labor expenses, pose significant barriers to their widespread adoption. However, the promising potential of these techniques should not be overlooked. Skin imaging technologies provide detailed visualization of skin layers and enable quantitative measurements of dermal thickness and elasticity, facilitating accurate tracking of treatment responses. Information obtained from photographs can be stored and analyzed multiple times. These quantitative results enhance comparability in longitudinal studies and clinical trials. Additionally, the non‐invasive nature of these techniques improves patient compliance and satisfaction. Despite these advantages, further economic evaluations are necessary to identify the most cost‐effective strategies and guide decision‐making processes. A balanced approach that combines subjective scales with quantitative techniques may optimize the trade‐off between cost and accuracy.

The alteration of biophysical parameters is closely associated with the severity of skin aging and can quantify subtle skin changes following treatment that precede visible improvements, which may not yet be detected by subjective scales or photography. Their primary strength lies in generating quantifiable, reproducible data on specific physiological properties. Specific parameters should be selected based on therapeutic goals. For example, a cutometer can be used to confirm increased skin elasticity following collagen‐stimulating therapy, while the melanin index can demonstrate improvements in pigmentation. Additionally, these tools are most effective when used in conjunction with, rather than as a replacement for, subjective evaluation outcomes. However, these parameters may be influenced by various intrinsic and extrinsic factors, including age, sex, race, season, and exercise [45]. In practice, these factors should be minimized as much as possible.

Due to variations in genetic and environmental factors, the manifestation of skin aging varies significantly across different skin types [46, 47]. Currently, there is no standardized method for follow‐up assessments, which complicates the comparison of results across studies. Several subjective scales have been adopted and validated for specific skin types; however, their suitability and generalizability require further validation [48]. For objective methods, large‐scale research is necessary to establish robust reference intervals across diverse racial and ethnic groups [49].

In conclusion, this review provides an overview of the available noninvasive tools for therapy monitoring of facial rejuvenation. Given the recent explosion of research and development of novel treatments for skin aging, it is imperative for physicians to comprehend the various assessment methods. When discussing treatment options with patients or proposing new therapies, the selection of appropriate measurement tools is essential. The ideal evaluation system must consider therapeutic goals, convenience, budget, accuracy, and patient perceptions.

## Author Contributions

All authors contributed to the study conception and design. Literature research, data analysis, and manuscript editing were performed by Shasha Zhao. Meng Wang and Xiaodong Lai contributed to data collection and analysis. Yan Yan was responsible for manuscript review and supervision.

## Ethics Statement

The authors have nothing to report.

## Consent

The authors have nothing to report.

## Conflicts of Interest

The authors declare no conflicts of interest.

## Data Availability

Data sharing not applicable to this article as no datasets were generated or analyzed during the current study.
